# Significance of pelvic lymph node dissection during radical prostatectomy in high-risk prostate cancer patients receiving neoadjuvant chemohormonal therapy

**DOI:** 10.1038/s41598-022-13651-x

**Published:** 2022-06-11

**Authors:** Hiromichi Iwamura, Shingo Hatakeyama, Takuma Narita, Yusuke Ozaki, Sakae Konishi, Hirotaka Horiguchi, Hirotake Kodama, Yuta Kojima, Naoki Fujita, Teppei Okamoto, Yuki Tobisawa, Tohru Yoneyama, Hayato Yamamoto, Takahiro Yoneyama, Yasuhiro Hashimoto, Chikara Ohyama

**Affiliations:** 1 Department of Urology, Hirosaki University Graduate School of Medicine, Hirosaki, Japan; 2 Department of Advanced Blood Purification Therapy, Hirosaki University Graduate School of Medicine, Hirosaki, Japan; 3 Department of Advanced Transplant and Regenerative Medicine, Hirosaki University Graduate School of Medicine, Hirosaki, Japan

**Keywords:** Cancer, Surgical oncology, Prostate

## Abstract

We aimed to determine the survival and staging benefit of limited pelvic lymph node dissection (PLND) during radical prostatectomy (RP) in high-risk prostate cancer (PC) patients treated with neoadjuvant chemohormonal therapy. We retrospectively analyzed 516 patients with high-risk localized PC (< cT4N0M0) who received neoadjuvant androgen-deprivation therapy plus estramustine phosphate followed by RP between January 2010 and March 2020. Since we stopped limited PLND for such patients in October 2015, we compared the surgical outcomes and biochemical recurrence-free survival (BCR-FS) between the limited-PLND group (before October 2015, n = 283) and the non-PLND group (after November 2015, n = 233). The rate of node metastases in the limited-PLND group were 0.8% (2/283). Operation time was significantly longer (176 vs. 162 min) and the rate of surgical complications were much higher (all grades; 19 vs. 6%, grade ≥ 3; 3 vs. 0%) in the limited-PLND group. The inverse probability of treatment weighting-Cox analysis revealed limited PLND had no significant impact on BCR-FS (hazard ratio, 1.44; P = 0.469). Limited PLND during RP after neoadjuvant chemohormonal therapy showed quite low rate of positive nodes, higher rate of complications, and no significant impact on BCR-FS.

## Introduction

Pelvic lymph node dissection (PLND) during radical prostatectomy (RP) for localized prostate cancer (PC) has been performed since the procedure was established in the 1980s^1^. Although PLND can accurately determine the nodal stage, its indications, optimal extent, and therapeutic benefits remain controversial because of the lack of randomized controlled trials (RCTs)^2^.

Previous retrospective studies suggested that PLND during RP may have survival benefits mainly because some patients with pathological proven lymph node metastasis did not relapse without adjuvant therapy^3–7^. Recently, two RCTs comparing the extended and the limited PLND have been reported. The results showed that extended PLND did not improve the biochemical recurrence-free survival (BCR-FS) compared with limited PLND^8,9^. These results indicated that there is no difference in prognosis based on the extent of PLND. However, RCTs comparing PLND from no PLND remain unavailable. Furthermore, there are no reports, including retrospective studies, evaluating the survival and staging benefits of PLND during RP in patients with high-risk PC who received neoadjuvant therapy.

In our institution, all patients with high-risk PC undergoing RP have been treated with neoadjuvant androgen-deprivation therapy (ADT) plus low-dose estramustine phosphate (EMP) and limited PLND^10,11^. Because the rate of positive nodes in those patients was less than 1%, we questioned the significance of limited PLND and stopped limited PLND during RP in October 2015. Thus, the aim of the present study was to assess the survival and staging benefits of limited PLND during RP on BCR-FS in patients with high-risk PC who received neoadjuvant ADT plus low-dose EMP by comparing the outcomes before and after the discontinuation of PLND.

## Results

### Baseline characteristics

We identified 574 patients with high-risk localized PC (< cT4N0M0) who have received neoadjuvant therapy followed by RP between January 2010 and March 2020. Of those, we excluded patients treated with other neoadjuvant regimens (n = 41) and those who discontinued low-dose EMP during the neoadjuvant period due to adverse events (n = 17). Finally, we included 516 patients with high-risk PC treated with neoadjuvant chemohormonal therapy (ADT plus low-dose EMP) followed by RP. Among them, 283 underwent limited PLND and 233 did not. The limited-PLND group had a significantly higher rate of biopsy International Society of Urological Pathology grade group 5 (56.2% vs. 45.5%, P = 0.022) and a significantly lower rate of cT3 than the non-PLND group (30.7% vs. 53.2%, P = 0.001). The median risk of lymph node invasion was 16%. The median duration of the neoadjuvant chemohormonal therapy was 8.3 months (Table 1).

**Table 1:** Baseline characteristics.

	Overall (n = 516)	Limited-PLND group (n = 283)	Non-PLND group (n = 233)	P-value
Median age, year (IQR)	68 (65–72)	68 (64–72)	69 (66–72)	0.040
ECOG-PS ≥ 1, n (%)	2 (0.4)	1 (0.4)	1 (0.4)	1.000
Anticoagulant use, n (%)	33 (6.4)	23 (8.1)	10 (4.3)	0.103
Median initial PSA, ng/mL (IQR)	9.3 (6.1–17.1)	9.1 (6.0–16.6)	9.4 (6.3–17.2)	0.698
**Biopsy Gleason score, n (%)**				< 0.001
6 (3 + 3; ISUP GG1)	13 (2.5)	3 (1.1)	10 (4.3)	
7 (3 + 4; ISUP GG2)	70 (13.6)	30 (10.6)	40 (17.2)	
7 (4 + 3; ISUP GG3)	40 (7.8)	14 (4.9)	26 (11.2)	
8 (4 + 4, 3 + 5, 5 + 3; ISUP GG4)	128 (24.8)	77 (27.2)	51 (21.9)	
9, 10 (4 + 5, 5 + 4, 5 + 5; ISUP GG5)	265 (51.4)	159 (56.2)	106 (45.5)	0.022
**Clinical tumor stage, n (%)**				< 0.001
cT1	151 (29.3)	98 (34.6)	53 (22.7)	
cT2	154 (29.8)	98 (34.6)	56 (24.0)	
cT3	211 (40.9)	87 (30.7)	124 (53.2)	< 0.001
Median risk of LNI (Briganti nomogram), % (IQR)	16 (8–37)	14 (7–32)	18 (8–46)	0.024
Risk of LNI < 5%, n (%)	43 (8.3)	27 (9.5)	16 (6.9)	0.337
Neoadjuvant therapy, n (%)	516 (100.0)	283 (100.0)	233 (100.0)	1.000
Median duration of neoadjuvant therapy, months (IQR)	8.3 (7.0–9.9)	8.1 (7.0–9.4)	8.5 (7.1–10.2)	0.018

*PLND* pelvic lymph node dissection, *IQR* interquartile range, *ECOG-PS* Eastern Cooperative Oncology Group Performance Status, *PSA* prostate-specific antigen, *ISUP GG* the International Society of Urological Pathology grade group, *LNI* lymph node invasion.

### Surgical and pathological outcomes

**Figure 1:**
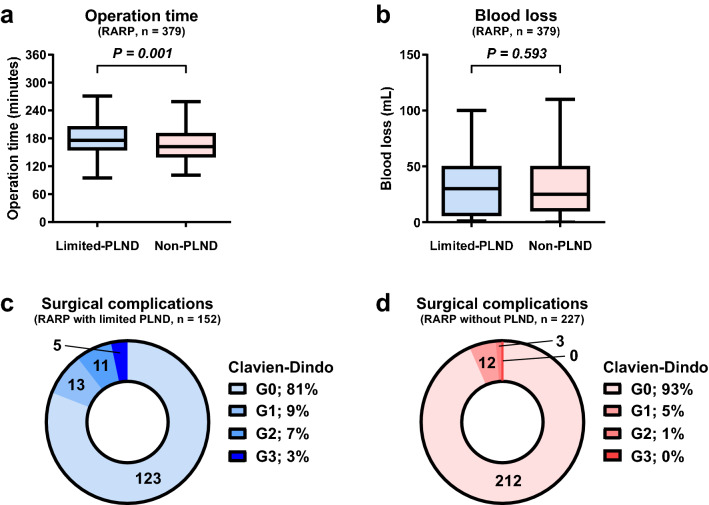
Operation time (**a**), blood loss (**b**), and surgical complications (**c,d**), between the limited-PLND and non-PLND groups (excluding RRP cases). *PLND* pelvic lymph node dissection, *RARP* robot-assisted radical prostatectomy, *RRP* retropubic radical prostatectomy. *Since surgical invasiveness and difficulty greatly differ between RRP and RARP, only RARP cases were included to examine the impact of PLND on surgical outcomes.

Considering the historical cohort study, the surgery type was significantly different between the groups; the rate of robot-assisted radical prostatectomy (RARP) was 53.7% and 97.4% in the limited-PLND and non-PLND groups, respectively (P < 0.001). Accordingly, the non-PLND group had a significantly longer median operation time (152 vs. 161 min, P = 0.001) and significantly lesser median blood loss (100 vs. 25 mL, P < 0.001) than the other group (Table 2). Since surgical invasiveness and difficulty greatly differ between retropubic radical prostatectomy (RRP) and RARP, we also analyzed the surgical outcomes only in RARP cases to examine the impact of PLND itself on operation time, blood loss, and surgical complications. In patients who received RARP, the operation time was significantly shorter in the non-PLND group than in the limited-PLND group (176 vs. 162 min, P = 0.001), but the blood loss was not significantly different between the groups (30 vs. 25 mL, P = 0.593). The incidence of surgical complications was significantly higher (all grades; 19 vs. 6%, P < 0.001, grade ≥ 3; 3 vs. 0%, P = 0.010) in the limited-PLND group (Fig. 1a–d, Table S1). The pathological tumor stage was not significantly different between the groups, but the rate of surgical margin positivity was significantly higher in the non-PLND group than in the limited-PLND group (6.0% vs. 11.6%, P = 0.027) (Table 2).

**Table 2:** Surgical and pathological outcomes.

	Overall (n = 516)	Limited-PLND group (n = 283)	Non-PLND group (n = 233)	*P*-value
**Surgery type, n (%)**				< 0.001
RARP	379 (73.4)	152 (53.7)	227 (97.4)	
RRP	137 (26.6)	131 (46.3)	6 (2.6)	
Median operation time, min (IQR)	156 (131–184)	152 (121–182)	161 (138–189)	0.001
Median blood loss, mL (IQR)	50 (20–243)	100 (25–735)	25 (10–50)	< 0.001
**Prostatectomy Gleason score, n (%)**				< 0.001
No residual tumor	55 (10.7)	29 (10.2)	26 (11.2)	
6 (3 + 3; ISUP GG1)	3 (0.6)	1 (0.4)	2 (0.9)	
7 (3 + 4; ISUP GG2)	12 (2.3)	1 (0.4)	11 (4.7)	
7 (4 + 3; ISUP GG3)	63 (12.2)	11 (3.9)	52 (22.3)	
8 (4 + 4, 3 + 5, 5 + 3; ISUP GG4)	21 (4.1)	19 (6.7)	2 (0.9)	
9, 10 (4 + 5, 5 + 4, 5 + 5; ISUP GG5)	362 (70.2)	222 (78.4)	140 (60.1)	< 0.001
**Pathological tumor stage, n (%)**				0.584
ypT0	55 (10.7)	29 (10.2)	26 (11.2)	
ypT2	314 (60.9)	168 (59.4)	146 (62.7)	
ypT3	147 (28.5)	86 (30.4)	61 (26.2)	0.327
Surgical margin positive, n (%)	44 (8.5)	17 (6.0)	27 (11.6)	0.027
Median number of dissected nodes, n (IQR)		4 (3–7)		
**Number of positive nodes, n (%)**				
0		281 (99.2)		
1		1 (0.4)		
2		1 (0.4)		
Biochemical recurrence, n	85	55	30	
Cancer-specific mortality, n	5	4	1	
All-cause mortality, n	13	10	3	
Median follow-up periods, months (IQR)	57 (31–81)	77 (63–94)	34 (20–46)	< 0.001

*PLND* pelvic lymph node dissection, *RARP* robot-assisted radical prostatectomy, *RRP* retropubic radical prostatectomy, *IQR* interquartile range, *ISUP GG* the International Society of Urological Pathology grade group.

**Figure 2:**
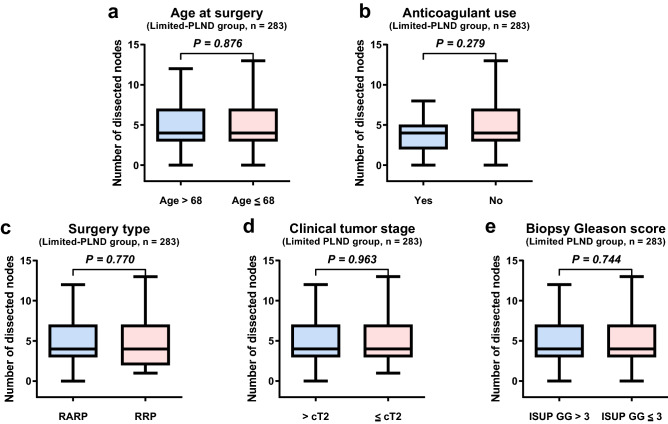
Number of dissected nodes stratified by age at surgery (**a**), anticoagulant use (**b**), surgery type (**c**), clinical tumor stage (**d**), and biopsy Gleason score (**e**) in the limited-PLND group. *PLND* pelvic lymph node dissection, *RARP* robot-assisted radical prostatectomy, *RRP* retropubic radical prostatectomy, *ISUP GG* the International Society of Urological Pathology grade group.

In the limited-PLND group, the median number of dissected nodes was 4 (interquartile range: 3–7) and the rate of positive nodes was 0.8% (2/283) (Table 2). Age, anticoagulant use, surgery type, clinical tumor stage, and biopsy Gleason score had no significant impact on the number of dissected nodes (Fig. 2a–e, Table S2).

### Oncological outcomes

**Figure 3:**
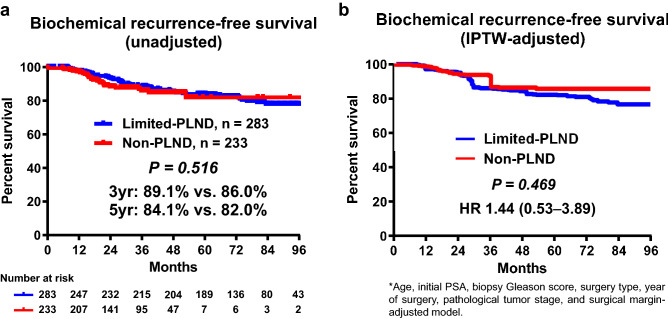
Unadjusted (**a**) and IPTW-adjusted biochemical recurrence-free survival (**b**), between the limited-PLND and non-PLND groups. *IPTW* inverse probability of treatment weighting, *PLND* pelvic lymph node dissection, *PSA* prostate-specific antigen.

The median follow-up period was significantly longer in the limited-PLND group than in the non-PLND group (77 vs. 34 months, P < 0.001). Accordingly, BCR was observed in 55 and 30 patients in the limited-PLND and non-PLND groups, respectively (Table 2). The unadjusted Kaplan–Meier analysis showed that BCR-FS was not significantly different between the groups (3 years, 89.1% vs. 86.0%; 5 years, 84.1% vs. 82.0%; P = 0.516, Fig. 3a). According to the multivariate Cox regression analysis, age, initial prostate-specific antigen (PSA), year of surgery, pathological tumor stage, and surgical margin were independent prognostic factors for BCR-FS (HR: 0.95, 1.02, 2.46, 2.49, and 2.06, respectively), whereas PLND was not (Table 3). Furthermore, the inverse probability of treatment weighting (IPTW)-adjusted Cox regression analysis, in which the potential confounders for BCR-FS (age, initial PSA, biopsy Gleason score, surgery type, year of surgery, pathological tumor stage, and surgical margin) were adjusted in both groups, showed that BCR-FS was not significantly different between the two groups (HR: 1.44; P = 0.469, Fig. 3b).

**Table 3:** Multivariate Cox regression analysis for biochemical recurrence-free survival in the overall cohort.

Factor		HR (95% CI)	*P*-value
Age	Continuous	0.95 (0.92–0.99)	0.011
Initial PSA	Continuous	1.02 (1.01–1.03)	0.001
Biopsy Gleason score	ISUP GG0–5	1.21 (0.98–1.50)	0.076
Surgery type	RARP vs RRP	0.85 (0.62–1.16)	0.310
Years of surgery	After vs before May 2015	2.46 (1.02–5.96)	0.046
Pathological tumor stage	ypT0–3	2.49 (1.62–3.85)	< 0.001
Surgical margin	Positive	2.06 (1.15–3.70)	0.016
PLND	Limited PLND vs no PLND	1.32 (0.58–2.98)	0.510

*HR* hazard ratio, *CI* confidence interval, *PSA* prostate-specific antigen, *ISUP GG* the International Society of Urological Pathology grade group, *RARP* robot-assisted radical prostatectomy, *RRP* retropubic radical prostatectomy, *PLND* pelvic lymph node dissection.

## Discussion

In this study, we evaluated the oncological benefits of limited PLND during RP in patients with high-risk PC treated with neoadjuvant ADT plus low-dose EMP, with adjustment for patients’ background, surgery type, and clinical and pathological disease status using IPTW-adjusted model. Compared with no PLND, limited PLND had no significant impact on BCR-FS. Furthermore, PLND was significantly associated with a longer operation time and higher rate of surgical complications, with only 0.8% positive nodes.

The survival benefit of PLND for malignancies has been debated in various types of cancer. Except colorectal cancers^12^, bladder^13^, esophageal^14^, gastric^15^, pancreatic^16^, lung^17^, breast^18^, and ovarian cancers did not benefit from PLND in RCTs^19^. Regarding PC, the survival benefit of PLND during RP had been discussed according to the results of retrospective studies. Previous retrospective studies demonstrated that PLND during RP is a staging procedure and may also have a positive impact on survival^3–7^. The main rationale for the survival benefit of PLND is that 24% of patients with positive lymph nodes did not relapse without adjuvant therapy^3^ and 30% of patients who underwent salvage LND for BCR and nodal recurrence had an undetectable PSA (< 0.1 ng/mL)^4^. Other studies argued that more extended PLND identified twice as many lymph node metastases as limited PLND, which may help to cure lymph node micrometastases^5–7^. The 2021 EAU guidelines strongly recommend extended PLND for intermediate-risk PC with an estimated risk for lymph node metastases of ≥ 5% and all high-risk PC for optimal nodal staging^20^. However, the 2017 AUA guidelines did not mention any recommendation because the survival benefits of extended PLND still have no supporting evidence^21^. A recent systematic review^22^ including 44 retrospective studies (n = 275,269) found that BCR-FS, metastatic-free survival, and cancer-specific survival were not significantly different between patients with PLND of any forms and those without PLND. Conversely, the more extensive the extent of PLND, the greater the adverse outcomes such as operating time, blood loss, postoperative complications, and hospitalization duration^22^. Furthermore, two recently published RCTs demonstrated that extended PLND had no survival benefits compared with limited PLND (HRs for BCR-FS: 0.91 and 1.04, respectively)^8,9^. Although these two RCTs revealed that the extent of PLND has no impact on survival outcomes, RCTs comparing PLND and no PLND, particularly in patients treated with intensive neoadjuvant chemohormonal therapy, remain unavailable. In the present study, limited PLND during RP after neoadjuvant chemohormonal therapy showed no significant impact on survival outcomes, with a significantly longer operation time and higher rate of postoperative adverse events than no PLND. Furthermore, only two patients (0.8%) had positive nodes, possibly because of the neoadjuvant chemohormonal therapy. Of the two patients, one had no BCR and the other had a PSA level of > 0.2 ng/mL immediately after surgery and died of PC 6 years later. Thus, of the 283 patients who underwent limited PLND, only one (0.4%) had a survival benefit from PLND and the others (99.6%) underwent unnecessary PLND, along with the higher rate of surgical complications. These results suggest that limited PLND during RP may be omitted in patients with high-risk PC who have received neoadjuvant chemohormonal therapy. An ongoing trial comparing extended PLND and no PLND (NCT03921996) will help us elucidate whether PLND during RP can really be omitted, although there are no ongoing trials to determine whether neoadjuvant therapy can eliminate PLND.

Generally, PLND during RP is performed in > 90% of patients with high-risk PC^23^, and the rate of positive nodes is 5–37% in patients without neoadjuvant therapy^24–28^. In a neoadjuvant setting, the rate of positive nodes is 0–30%, with various regimens of neoadjuvant therapy^29^. Although guidelines do not recommend any neoadjuvant therapies outside clinical trials for patients who have elected to undergo RP^20,21^, neoadjuvant therapy is associated with a decreased rate of pT3, a reduced rate of positive surgical margin, and a lower incidence of lymph node metastases^30^. In a recent meta-analysis, only regimens of neoadjuvant therapy comprising EMP significantly reduced the rate of positive nodes (odds ratio 0.05–0.11)^10,11,24,29^. Similarly, the rate of positive nodes in the present study was only 0.8% (2/283) despite the relatively high estimated risk of lymph node invasion (median, 16%). These results suggest that PLND is not beneficial even for nodal staging in patients with high-risk PC who received neoadjuvant chemohormonal therapy containing EMP. Nevertheless, selecting the optimal candidate for PLND remains necessary because some patients may still benefit from PLND with or without neoadjuvant therapy. The Briganti nomogram is the most common tool used to determine the indication for PLND during RP^31^. With a 5% nomogram cutoff, 66% of patients will be spared from PLND. However, even with this nomogram, 27% of patients will still undergo unnecessary PLND (patients with a nomogram-derived lymph node metastasis risk of ≥ 5% without histologic lymph node invasion). Additionally, nomogram cannot be applied in the neoadjuvant setting. Meanwhile, novel imaging modalities such as whole-body magnetic resonance imaging^32,33^ and prostate-specific membrane antigen–targeted positron emission tomography (PSMA-PET)^34,35^ can differentiate patients with cN1 from those diagnosed with cN0 by conventional imaging. Ongoing trials (NCT04832958 and NCT04457245) for image-guided treatment protocols for localized PC using PSMA-PET will provide us with a novel treatment strategy for PLND during RP.

Considering that surgery is complex and surgeon-dependent, some biases in surgical research need to be considered, even in RCTs^36^. Surgeons may change the indication and extent of PLND according to the patients’ background, disease status, and surgery type (selection and performance biases). Although this is a retrospective study with various surgeons, selection bias was minimized because the indication of PLND relied on the treatment strategy in our institution and not on the surgeons (all patients with high-risk PC underwent limited PLND before October 2015 and did not after November 2015). The IPTW analysis also contributed to minimizing the selection bias. Furthermore, all patients in the limited PLND group underwent the same lymphadenectomy technique. The number of lymph nodes dissected was not affected by patients’ age, anticoagulant use, surgery type, clinical tumor stage, and biopsy Gleason score. Therefore, the present study has minimal selection bias and performance bias despite its retrospective design. To evaluate external validity, a multicenter retrospective study including our affiliated hospitals that follow the same treatment strategy as ours for PLND is warranted.

This study has some limitations. First, retrospective design, limited sample size, and relatively shorter follow-up periods prevented us from attaining a definitive conclusion. Second, the quite low rate of positive nodes may have been due not only to intensive neoadjuvant therapy but also to inadequate PLND. Third, it is unclear whether the same results would be obtained with docetaxel or novel anti-androgens; the use of these drugs may further reduce the significance of lymph node dissection. Fourth, the follow-up period was significantly shorter in the non-PLND group because of the historical cohort design, which may introduce a selection bias. Finally, our data cannot deny the benefits of extended PLND after neoadjuvant chemohormonal therapy. Despite these limitations, our study is the first to reveal that limited PLND during RP after neoadjuvant chemohormonal therapy in high-risk PC patients does not improve BCR-FS with negligible staging benefit. Further research is needed to validate the present observation.

## Conclusion

Limited PLND after neoadjuvant chemohormonal therapy containing EMP showed no significant impact on BCR-FS, with quite low rate of positive nodes. Thus, limited PLND during RP may be omitted in patients with high-risk PC who have received neoadjuvant chemohormonal therapy.

## Methods

### Study design and participants

We retrospectively analyzed consecutive patients with high-risk localized PC (< cT4N0M0) treated with neoadjuvant ADT plus low-dose EMP followed by RP at Hirosaki University Hospital between January 2010 and March 2020. We collected information regarding patients’ background, disease status, and surgical, pathological, and oncological outcomes from their medical records. Patients with any missing information were excluded. We determined the tumor stage using the 2017 American Joint Committee on Cancer Staging Manual^37^. We defined high-risk PC as follows: initial PSA levels of ≥ 20 ng/mL, a biopsy Gleason score of ≥ 8 (International Society of Urological Pathology grade group ≥ 4), and/or clinical tumor stage T2c or T3 according to the D' Amico risk stratification^38^.

### Treatment procedures

For 6–9 months before RP, all patients with high-risk PC who have elected to undergo RP received neoadjuvant ADT (luteinizing hormone–releasing hormone agonist or gonadotropin-releasing hormone antagonist) plus low-dose EMP (280 mg/day) in our institution, as previously described^10,11,24^. With the approval of RARP in Japan in January 2012, RARP has been performed in most cases; in earlier cases, RRP was performed. Considering that the rate of positive lymph nodes was approximately 1% in patients with high-risk PC treated with neoadjuvant ADT plus low-dose EMP followed by RP at our institution^24^, we stopped PLND in October 2015. Thus, we categorized patients into two groups according to treatment strategy shift: limited-PLND group (patients with high-risk PC who received limited PLND; between January 2010 and October 2015) and non-PLND group (patients who did not receive limited PLND; after November 2015). The limited-PLND group underwent the same lymphadenectomy method, including removal of the bilateral obturator nodes. Surgical complications were evaluated using the Clavien–Dindo classification^39^. The risk of lymph node invasion was retrospectively calculated using the Briganti nomogram^31^.

### Pathological analysis

All prostatectomy specimens were sectioned using the whole-mount technique and then evaluated according to the International Society of Urological Pathology 2005/2014 guidelines^40,41^. Each dissected lymph node was cut into 3-mm slices, embedded separately in paraffin, stained with hematoxylin and eosin, and examined under the microscope. The results were described as the total number of lymph nodes dissected and the number of positive lymph nodes. An expert urological pathologist reviewed all biopsy and surgical specimens.

### Follow-up

Postoperatively, serum PSA and testosterone levels were evaluated in all patients every 3 months. These patients did not undergo adjuvant ADT or radiation therapy in our institution regardless of the pathological results. We defined the date of BCR as the date when the serum PSA level exceeded 0.2 ng/mL. If the PSA level did not decrease to less than 0.2 ng/mL postoperatively, the date of RP was considered as the date of BCR.

### Endpoints

The primary endpoint was evaluating the therapeutic effect of limited PLND on BCR-FS. The secondary endpoints were comparing surgical outcomes and complications between the groups, and the benefit of limited PLND in terms of nodal staging.

### Statistical analysis

All data were analyzed using R 4.0.2 (The R Foundation for Statistical Computing, Vienna, Austria; https://www.r-project.org/) and GraphPad Prism 9.00 (GraphPad Software, San Diego, CA, USA; https://www.graphpad.com/scientific-software/prism/). We compared categorical variables using the Fisher’s exact test or χ^2^ test. Quantitative variables were expressed as median with an interquartile range. The student’s *t*-test or the Mann–Whitney *U* test was used to compare differences between groups. Using the Kaplan–Meier method, we compared the BCR-FS between the limited-PLND and non-PLND groups. Hazard ratios (HR) with 95% confidence intervals (CIs) were obtained by multivariate Cox regression analysis using the IPTW-adjusted model. After creating a pseudopopulation, we used the propensity score–based IPTW method to remove the background imbalances between the groups and estimate the average treatment effect in an unbiased manner^42^. Multivariate IPTW-Cox analysis included the following steps. First, we calculated the propensity score using the parameters including age, initial PSA, biopsy Gleason score (we did not use pathological Gleason score that was affected by neoadjuvant chemohormonal therapy), surgery type, year of surgery (we divided into two groups in the middle of the entire period; before and after May 2015), pathological tumor stage, and surgical margin, for the limited-PLND group through logistic regression analysis. Second, we calculated the IPTW by the inverse probability of the “given” exposure. The treatment weights for the limited-PLND and non-PLND groups were determined using the following formula: 1/propensity score and 1/(1 − propensity score), respectively. Third, we performed multivariable Cox regression analysis with robust adjustment including two factors: treatment selection (limited-PLND group = 1) and treatment weighting. P-values of < 0.05 were considered statistically significant.

### Ethics approval statement

This retrospective study was approved by the Ethics Committee of Hirosaki University (2019-099). All participants had previously provided written informed consent for other clinical studies. Since all the data used in this study were obtained from the medical records, additional informed consent for this study was waived with approval by the Ethics Committee of Hirosaki University. The clinical and research activities being reported are consistent with the Principles of the Declaration of Helsinki.

## Supplementary Information

Supplementary Tables.
