# Supplementary material for: Significance of pelvic lymph node dissection during radical prostatectomy in high-risk prostate cancer patients receiving neoadjuvant chemohormonal therapy
Source: Sci Rep. 2022 Jun 11;12:9675. doi: 10.1038/s41598-022-13651-x (PMC9188590; doi:10.1038/s41598-022-13651-x)
Supplement: Supplementary file 1 — Supplementary Tables. [file 41598_2022_13651_MOESM1_ESM.docx]

Table S1. Surgical outcomes and complications between the limited and the non-PLND groups (excluded RRP cases)

|  | Limited-PLND group  (n = 152) | Non-PLND group  (n = 227) | *P-value* |
| --- | --- | --- | --- |
| Median operation time, min (IQR) | 176 (155–205) | 162 (141–191) | *0.001* |
| Median blood loss, mL (IQR) | 30 (7–50) | 25 (10–50) | *0.593* |
| Surgical complications (Clavien-Dindo classification) |  |  |  |
| All grades, n (%) | 29 (19) | 15 (7) | *<0.001* |
| Grade 1, n (%) | 13 (9) | 12 (5) |  |
| Grade 2, n (%) | 11 (7) | 3 (1) |  |
| Grade 3, n (%) | 5 (3) | 0 (0) | *0.010* |
| Grade ≥ 4, n (%) | 0 (0) | 0 (0) |  |

Table S2. Number of dissected nodes stratified by patients’ background, surgery type, and preoperative disease status

|  | Number of dissected nodes | | *P-value* |
| --- | --- | --- | --- |
|  | Median (IQR) | Average (min to max) |  |
| Age at surgery |  |  | *0.876* |
| > 68 | 4 (3–7) | 5 (0–12) |  |
| ≤ 68 | 4 (3–7) | 5 (0–18) |  |
| Anticoagulant use |  |  | *0.279* |
| Yes | 4 (3–5) | 4 (0–12) |  |
| No | 4 (3–7) | 5 (0–17) |  |
| Surgery type |  |  | *0.770* |
| RARP | 4 (3–7) | 5 (1–16) |  |
| RRP | 4 (2–7) | 5 (0–18) |  |
| Clinical tumor stage |  |  | *0.963* |
| > cT2 | 4 (3–7) | 5 (1–16) |  |
| ≤ cT2 | 4 (3–7) | 5 (0–18) |  |
| Biopsy Gleason score |  |  | *0.744* |
| ISUP GG > 3 | 4 (3–7) | 5 (0–18) |  |
| ISUP GG ≤ 3 | 4 (3–7) | 5 (0–16) |  |
